# Exposure Risk for Infection and Lack of Human-to-Human Transmission of *Mycobacterium ulcerans* Disease, Australia

**DOI:** 10.3201/eid2305.160809

**Published:** 2017-05

**Authors:** Daniel P. O’Brien, James W. Wynne, Andrew H. Buultjens, Wojtek P. Michalski, Timothy P. Stinear, N. Deborah Friedman, Andrew Hughes, Eugene Athan

**Affiliations:** Médecins Sans Frontières, London, UK (D.P. O’Brien);; University of Melbourne, Melbourne, Victoria, Australia (D.P. O’Brien, A.H. Buultjens, T.P. Stinear);; Barwon Health, Geelong, Victoria, Australia (D.P. O’Brien, N.D. Friedman, A. Hughes, E. Athan);; Commonwealth Scientific and Industrial Research Organisation, Geelong (J.W. Wynne, W.P. Michalski);; Deakin University School of Medicine, Geelong (E. Athan)

**Keywords:** Mycobacterium ulcerans, bacteria, tuberculosis and other mycobacteria, Mycobacterium ulcerans disease, Buruli ulcer, epidemiology, infection, exposure risk, genome sequencing, transmission family clusters, human-to-human transmission, Australia

## Abstract

We conducted epidemiologic and genetic analyses of family clusters of *Mycobacterium ulcerans* (Buruli ulcer) disease in southeastern Australia. We found that the incidence of *M. ulcerans* disease in family members was increased. However, the risk for exposure appeared short-term and not related to human-human transmission.

*Mycobacterium ulcerans* is a slow-growing organism that causes necrotizing infections of skin and soft tissue, often requiring reconstructive surgery and resulting in long-term disability (*1*,*2*). Prevailing opinion is that humans are infected from the environment; insects, such as mosquitoes (*3*,*4*), and water-residing biting arthropods (*5*,*6*), have been proposed as vectors for transmission. In Victoria, Australia, there is evidence that native opossums might be involved in transmission (*7*). However, despite extensive research, the environmental reservoir of the organism and mode of transmission remain unknown.

We postulated that examination of *M. ulcerans* disease (Buruli ulcer) family clusters might provide useful new information about disease epidemiology. Theoretically, genetically related first-degree relatives have similar susceptibility to disease, and families share the same environment and therefore a similar exposure risk. Thus, we examined the epidemiology of *M. ulcerans* disease in family clusters managed in a large prospective observational cohort from the Bellarine Peninsula in southeastern Australia. We used data collected from all confirmed *M. ulcerans* cases managed during January 1, 1998–April 12, 2016, at Barwon Health, a tertiary referral hospital in Geelong, Australia (*8*).

## The Study

For this study, only initial *M. ulcerans* lesions were analyzed. A family cluster was defined as multiple family members independently given a diagnosis of *M. ulcerans* disease who were living at the same residence at the time of diagnosis. Data was collected by using Epi Info 6 (Centers for Disease Control and Prevention, Atlanta, GA, USA) and analyzed by using Stata 12 (StataCorp LLC, College Station, TX, USA).

To determine the genetic relatedness of isolates derived from family clusters, we performed whole-genome sequencing and single-nucleotide polymorphism (SNP) analysis for 6 isolates derived from 3 family cluster pairs (Tables 1, 2). We sequenced DNA as 300-bp paired-end reads by using an MiSeq Sequencer (Illumina, Inc., San Diego, CA, USA). Resulting reads were mapped against the *M. ulcerans* Agy99 genome (*9*), including plasmid pMUM001 (*10*), by using Bowtie2 (*11*). Raw sequence reads for the 6 isolates have been deposited in the National Center for Biotechnology Information (Bethesda, MD, USA) Sequence Read Archive under BioProject accession no. PRJNA321660. We also performed whole-genome SNP analysis for 6 additional unrelated previously sequenced human *M. ulcerans* isolates (Sequence Read Archive accession no. SRP004497) obtained from the same disease-endemic region.

**Table 1:** Characteristics of 21 patients associated with family clusters of *Mycobacterium ulcerans* disease, Bellarine Peninsula, Victoria, Australia, 1998–2016*

Cluster	Isolate	Date of diagnosis	Time between lesions, mo	Location	Relationship	Patient age at diagnosis, y,/sex	Site of lesion	Type of lesion	WHO stage
1a	mu179	2008 Jul 21	0.4	PTL	Mother	54/F	Right thigh	Ulcer	1
1b	mu180	2008 Aug 4		PTL	Daughter	26/F	Left calf	Ulcer	1
2a	mu248	2010 Oct 24	20.6	PTL	Husband	84/M	Right forearm	Ulcer	1
2b	mu394	2012 Jul 4		PTL	Wife	84/F	Right forearm	Ulcer	1
3a	NT	2011 Jul 25	0.1	QUE	Husband	76/M	Right ankle	Ulcer	3
3b	NT	2011 Jul 28		QUE	Wife	75/F	Right elbow	Ulcer	1
4a	mu294	2011 Aug 22	1.3	PTL	Wife	65/F	Right knee	Ulcer	1
4b	mu308	2011 Sep 29		PTL	Husband	65/M	Left calf	Ulcer	1
5a	NT	2011 Aug 25	1.1	BH	Father	56/M	Right leg	Ulcer	1
5b	NT	2011 Sep 26		BH	Son	26/M	Right leg	Ulcer	1
6a	NT	2012 Jun 19	22.7	PTL	Wife	34/F	Left knee	Ulcer	1
6b	NT	2014 Apr 30		PTL	Husband	37/M	Right ankle	Ulcer	1
7a	NT	2012 Aug 14	22.9	QUE	Wife	74/F	Left ankle	Ulcer	1
7b	NT	2014 Jul 3		QUE	Husband	76/M	Left leg	Ulcer	1
8a	NT	2012 Oct 16	15.9	BH	Sister	20/F	Right foot	Ulcer	1
8b	NT	2014 Feb 14		BH	Brother	18/M	Left leg	Ulcer	1
9a	NT	2013 Apr 27	12.7	QUE	Wife	85/F	Right ankle	Ulcer	1
9b	NT	2014 May 12		QUE	Husband	90/M	Left forearm	Ulcer	1
10a	NT	2013 Dec 10	2.8	PTL	Father	34/M	Left hand	Ulcer	1
10b	NT	2014 Mar 4		PTL	Daughter	4/F	Right knee	Nodule	1
10c	NT	2014 Mar 5	0.0	PTL	Son	7/M	Right ankle	Nodule	1

*BH, Barwon Heads; NT, not tested; PTL, Point Lonsdale; QUE, Queenscliff; WHO, World Health Organization.

**Table 2:** Description of 8 single-nucleotide polymorphisms specific to >1 of 6 family cluster isolates of *Mycobacterium ulcerans* disease, Bellarine Peninsula, Victoria, Australia, 1998–2016*

Position	Loci	Protein	Substitution	Amino acid change	Isolate	Coverage statistics
398430	Intergenic	–	G/A	–	mu179	T: 0, A: 35, G: 0, C: 1
398430	Intergenic	–	G/A	–	mu180	T: 0, A: 67, G: 0, C: 0
398430	Intergenic	–	G/A	–	mu248	T: 0, A: 100, G: 0, C: 1
398430	Intergenic	–	G/A	–	mu294	T: 0, A: 75, G: 0, C: 0
398430	Intergenic	–	G/A	–	mu308	T: 0, A: 58, G: 0, C: 0
1758272	MUL_1618	Membrane protein	C/T	Synonymous	mu248	T: 91, A: 1, G: 0, C: 0
2153447	MUL_1947	Thiamine pyrophosphate	A/G	Lys→Arg	mu294	T: 0, A: 1, G: 58, C: 0
2153447	MUL_1947	Thiamine pyrophosphate	A/G	Lys→Arg	mu308	T: 0, A: 0, G: 40, C: 0
2462577	MUL_2205	Hypothetical protein	T/C	Asp→Gly	mu179	T: 1, A: 1, G: 0, C: 47
4359638	MUL_3902	Membrane protein	C/A	Ala→Ser	mu180	T: 0, A: 60, G: 1, C: 0
4359638	MUL_3902	Membrane protein	C/A	Ala→Ser	mu248	T: 0, A: 108, G: 0, C: 1
5189291	Intergenic	–	G/T	–	mu248	T: 76, A: 0, G: 4, C: 0
5354966	MUL_4830	Putative GTPase	T/C	Synonymous	mu180	T: 2, A: 0, G: 1, C: 18
5354966	MUL_4830	Putative GTPase	T/C	Synonymous	mu248	T: 0, A: 0, G: 0, C: 20
5577431	MUL_5032	Immunogenic protein mbt64	A/G	Synonymous	mu394	T: 0, A: 0, G: 28, C: 0

*A total of 4,918 core single-nucleotide polymorphisms were identified for all 6 isolates compared with the African Agy99 reference genome. –, not applicable (mutations were not within a coding region).

A total of 324 patients with *M. ulcerans* disease from the Bellarine Peninsula, Victoria, Australia, were managed in the Barwon Health observational cohort during January 1, 1998–April 12, 2016. Median age was 57 years (IQR 34–74 years), and 164 patients (50.6%) were men. For the whole cohort, a combined time of 1,968.5 years had elapsed from diagnosis of the initial *M. ulcerans* lesions until the time of study analysis (April 12, 2016). The median duration elapsed from initial diagnosis until study analysis was 4.7 years (IQR 2.8–9.7 years).

**Figure 1:**
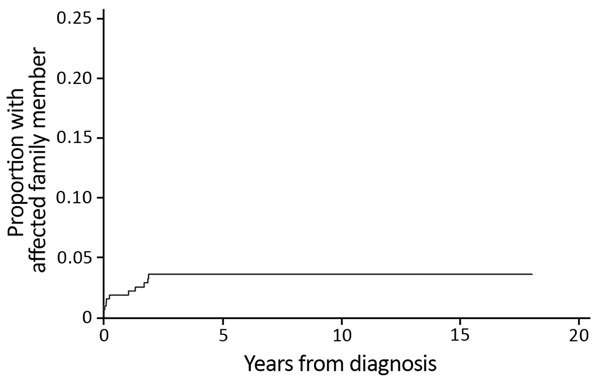
Cumulative proportion of patients with a family member affected by *Mycobacterium ulcerans* disease, Barwon Health cohort, Bellarine Peninsula, Victoria, Australia, 1998–2016.

Twenty-one (6.5%) patients were part of a family cluster (Table 1), 9 genetically related and 12 related by marriage. All family clusters were diagnosed after the beginning of 2008. We found that significantly fewer family clusters were diagnosed during the first half of the study period (0 of 92 cases during 1998–2007) than in the second half (21 of 232 cases during 2008–2016) (p<0.01). The median time between diagnoses of *M. ulcerans* lesions in an additional family member, after the initial family member was given a diagnosis, was 2.8 months (IQR 1.1–20.6 months). The rate of new diagnosis of an *M. ulcerans* lesion in another family member was 5.69/1,000 person-years (95% CI 3.15–10.29/1,000 person-years). We determined the cumulative proportion of patients given a diagnosis who had an affected family (Figure 1).

Core SNPs based on common variable nucleotide positions were identified for the 6 examined family isolates by whole-genome sequencing. A total of 4,918 core SNPs ascribed to the African Agy99 reference genome were identified according to strict filtering criteria. Only 8 SNPs were specific to >1 of the 6 isolates (Table 2). Of the 8 SNPs that differed among the isolates, only 3 were nonsynonymous substitutions. The remaining 5 SNPs were either intergenic or synonymous mutations.

**Figure 2:**
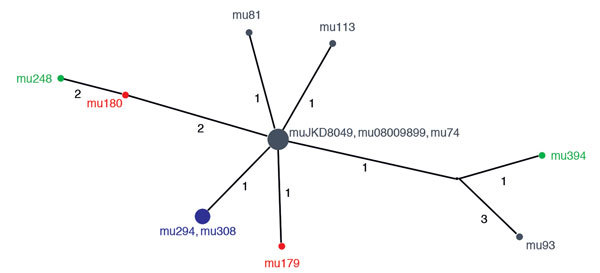
Median joining network of 12 SNPs of 12 *Mycobacterium ulcerans* isolates from patients with *Mycobacterium ulcerans* disease, Barwon Health cohort, Bellarine Peninsula, Victoria, Australia, 1998–2016. Node colors indicate clusters. Blue, cluster 4a/4b; red, cluster 1a/1b; green, cluster 2a/2b. Black nodes represent 6 unrelated isolates. The size of each node is proportional to the number of genetically identical isolates with identical genotypes. Values indicate number of SNPs between each node. SNP, single-nucleotide polymorphism.

Pairwise comparisons of family cluster isolates showed that isolates from the 4a/4b pair were genetically identical. In contrast, isolates from the 2a/2b and 1a/1b pairs contained several isolate-specific SNPs (Table 2; Figure 2). SNP analysis of unrelated *M. ulcerans* isolates from the same disease-endemic area showed that 3 of the 6 isolates were also genetically identical (Figure 2), which demonstrated that unrelated isolates can share a common genotype. The remaining 3 isolates contained 1–3 unique SNPs. Thus, family cluster isolates were not any more closely genetically related than 6 random isolates from the same geographic region.

## Conclusions

Our examination of family clusters of *M. ulcerans* disease provides useful insights into the environmental reservoir and mode of transmission of this organism. First, the median time to diagnosis between family members was short (2.8 months), and no family members were given a diagnosis of an *M. ulcerans* lesion >23 months apart in a cohort spanning 18 years and nearly 2,000 combined years of elapsed time since diagnosis. This finding suggests that family members have been exposed to a source in the family’s environment that persists only for a short period.

Second, with an incubation period for *M. ulcerans* disease estimated to be a median of 4.5 months (*12*), the observation that the median time between diagnoses in family clusters was <3 months suggest that infections were not being transmitted between family members. Further evidence against human-to-human transmission is apparent from whole-genome SNP analysis, which showed that pairs of isolates from 2 (2a/2b and 1a/1b) of 3 family clusters were not genetically identical. These findings support previous suggestions that *M. ulcerans* is unlikely to be transmitted from person to person (*13*).

Unknown is the type of short-term exposure that leads to the close temporal relation of family clustered infections. Opossums have been proposed as a source, either through contamination of the environment by infected feces or by an intermediate vector, such as mosquitoes, which transfer the infection from infected opossums to humans by a bite (*7*). Infected opossum(s) in the family environment might cause cases of human infection, then subsequently die of the disease (*14*), removing the source of infection. Alternatively, transmission could be related to a short-term change in the environment involving soil or foliage as a result of such events as home construction and renovation, or planting and removing trees or grasses (*13*). Mosquitoes in the area might be transiently infected/contaminated with *M. ulcerans* and infect humans through bites during this time (*15*).

In summary, the incidence rate of lesions in another family member (5.69/1,000 person-years) was higher than reported incidence rates during 2005–2009 in the general population of the Bellarine Peninsula (0.85–4.04 cases/year/1,000 population) (*7*). This finding suggests that genetic susceptibility or, more likely, localized exposure risk increases the likelihood of infection.

The incidence of *M. ulcerans* disease family clusters in an observational cohort in southeastern Australia was higher than in the general population of the disease-endemic area. However, when clusters occur, they are closely temporally related, which suggests a short-term risk for exposure and infection. Epidemiologic and genetic evidence suggests human-to-human transmission is not the source of infection.
